# Seasonal and fasting induced changes in iron metabolism in Djungarian hamsters

**DOI:** 10.1371/journal.pone.0293971

**Published:** 2023-11-06

**Authors:** Rawan Kawach, Victoria Diedrich, Andreas Gruber, Kerstin Leopold, Annika Herwig, Maja Vujić Spasić

**Affiliations:** 1 Institute of Comparative Molecular Endocrinology, Ulm University, Ulm, Germany; 2 Institute of Neurobiology, Ulm University, Ulm, Germany; 3 Institute of Analytical and Bioanalytical Chemistry, Ulm University, Ulm, Germany; Universidade de Brasilia, BRAZIL

## Abstract

Djungarian hamsters are small rodents that show pronounced physiological acclimations in response to changes in photoperiod, and unfavorable environmental conditions such as reduced food availability and low external temperature. These include substantial adjustments, such as severe body weight loss and the use of daily torpor. Torpor is a state of decreased physiological activity in eutherms, usually marked by low metabolic rate and a reduced body temperature. In this study, we investigated the effects of photoperiodic acclimation and food deprivation on systemic iron metabolism in Djungarian hamsters. Our study illustrates the association between liver iron levels and the incidence of torpor expression during the course of the experiment. Moreover, we show that both, acclimation to short photoperiods and long-term food restriction, associated with iron sequestration in the liver. This effect was accompanied with hypoferremia and mild reduction in the expression of principal iron-hormone, hepcidin. In addition to iron, the levels of manganese, selenium, and zinc were increased in the liver of hamsters under food restriction. These findings may be important factors for regulating physiological processes in hamsters, since iron and other trace elements are essential for many metabolic and physiological processes.

## Introduction

Djungarian hamsters (or Siberian hamsters, *Phodopus sungorus*) are small rodents that inhabit southwestern Siberia and northeastern Kazakhstan. The hamsters show pronounced physiological acclimatizations in the anticipation of harsh continental winter. As the day length decreases, the animals reduce food intake and body weight, halt reproduction, and develop a white and insulating winter fur [1,2]. After largely completing these adjustments, they start to naturally express spontaneous daily torpor. Together, these acclimatizations enable the animals to survive periods of energetically unfavorable environmental conditions such as reduced food and water availability combined with low external temperature [3]. All physiological acclimatizations are ultimately induced by the shortened photoperiod that is translated into a hormonal signal via the photoneuroendocrine pathway [3,4].

Daily torpor defines a state of decreased physiological activity in mammals and birds that is characterized by reduced metabolic rate for several hours [1,2,5] and a transient decrease in body temperature from the normothermic value of ∼37°C to values below 32°C for less than 24 hours [3,5–8]. Hamsters show highly individual responses to the switch from a long to a short photoperiod, whereby a certain percentage of animals does not undergo acclimation and/or torpor expression [9]. Reduced food intake and consequent reduction of body weight seem to play a significant role in the initiation and manifestation of torpor [10]. This becomes evident from the studies showing that daily torpor episodes can be triggered by long-term food restriction paradigms in long photoperiod [10,11,12].

The circadian clock appears to be involved in torpor control [7,13,14]. A study by Crawford *et* al. reported torpor-associated changes in the expression of several clock genes (such as *Per1*, *Per2*, *Bmal1*, *Cry2*, *Dbp*, and *Rev-erbα*) in heart, lung, and the liver [14]. Interestingly, the same study reported changes in the expression of two iron genes: the iron-exporter ferroportin was downregulated in short photoperiod-acclimated hamsters after arousal from daily torpor, while the transferrin receptor, which mediates cellular iron uptake, was upregulated [14]. Although these data suggest potential changes in cellular iron levels during daily torpor, the role of iron is still not clear, neither regarding the anticipatory long-term seasonal acclimation and the short-term acclimation to acute energy shortage, nor regarding the physiologically challenging expression of daily torpor.

Iron is an essential trace element involved in numerous fundamental cellular processes in the body, including DNA synthesis, cellular respiration, energy production, and cell growth and death [15]. Moreover, iron is the main component of hemoglobin and as such is required for oxygen transport within red blood cells [15]. The levels of iron in the body must be tightly regulated to prevent iron overload and iron deficiency [16]. The principal regulator of iron metabolism is the small peptide hormone hepcidin, which is synthesized in the liver and secreted into the blood [17]. Hepcidin acts on the iron-exporter protein ferroportin leading to its degradation [18]. By doing so, hepcidin controls the rate of iron absorption in the duodenum, the iron release from the macrophages, and the iron accumulation in the liver [19]. Importantly, the metabolism of iron and its homeostasis are controlled by a variety of mechanisms that are highly conserved throughout the mammalian kingdom [20].

We provide here the first insights into the status of iron and several other trace elements in the Djungarian hamster as an important model organism for the investigation of seasonality and daily torpor. Our data highlight iron sequestering in the liver of short photoperiod-acclimated hamsters expressing spontaneous torpor bouts and of long photoperiod-acclimated hamsters expressing forced, fasting-induced torpor. We postulate that this effect may be related to protective mechanisms as vital part of energy-saving seasonal and acute acclimation processes.

## Material and methods

### Animals

Djungarian hamsters (*Phodopus sungorus*) were born and kept in the laboratory breeding facility of the Institute of Neurobiology, University of Ulm, Germany. From weaning on, the animals were housed in individual cages equipped with wood shavings and cellulose bedding (Makrolon type III, area: 820 cm^2^) under an artificial long photoperiod (LP) with 16 h of light and 8 h of darkness (16:8) at a constant ambient temperature of 19±2°C and a relative humidity of 40±10%. All hamsters received food (Altromin Hamster Breeding Diet 7014, Lage, Germany) and water *ad libitum*. Supplementary feeding of sunflower seed, oat flakes, and cucumber was provided once a week. Animal husbandry and all experiments performed at the University of Ulm were in accordance with the German Animal Protection Law and were approved by the regional council in Tübingen, Germany (licence number 1411 and 1432).

### Acclimation to short photoperiod (SP-AL)

**Fig 1:**
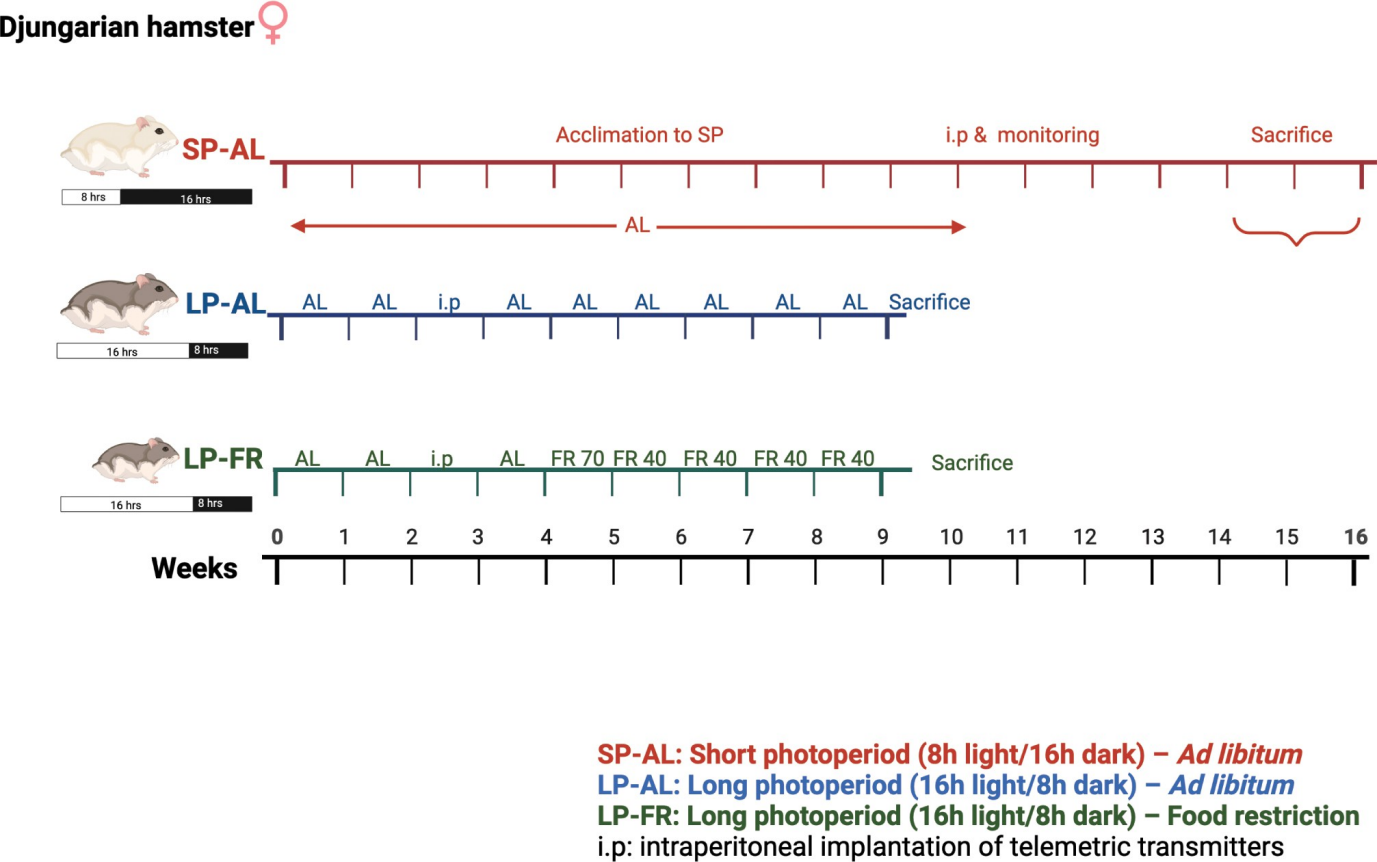
Scheme of animal experiment protocol in the present study. First group of Djungarian hamsters (*n* = 17) was exposed to a short photoperiod (SP) with 8h light and 16h dark and received standard food *ad libitum* (AL). The second group of hamsters was subjected to a long photoperiod under *ad libitum* (LP; 16h light and 8h dark; *n* = 10) or subjected to food restriction (FR) for 5 weeks in a long photoperiod (LP; 16h light and 8h dark; *n* = 10). For the experimental details please refer to the Material and Methods.

A group of 17 female Djungarian hamsters (9±2 months of age) was transferred to an artificial short photoperiod (SP) with 8 h of light and 16 h of darkness (8:16) to induce winter acclimation and spontaneous daily torpor expression (see Fig 1). All animals received food and water *ad libitum* throughout the experiment; supplemental feeding was maintained. The degree of acclimation was monitored by weekly assessment of body mass and fur index according to Figala et al [21] until the end of experiment. An index of 1 accounted for grey-brown summer fur and 6 for a white, well-insulating winter fur. After 10–12 weeks in SP, all hamsters were intraperitoneally implanted with telemetric transmitters (TA11TA-F10, Data Science International, St Paul, MN, USA; silicone-coated, 1.1 cc volume, 1.6 g weight, 0.15°C accuracy) to continuously measure body temperature as thus to record spontaneous daily torpor bouts (Fig 1) (for details see [3,11]). The surgery was carried out under carprofen analgesia (5mg/kg; Rimadyl®, Zoetis Deutschland GmbH, Berlin) and isoflurane anaesthesia (2.5% induction, 0.75–2.0% maintenance; Forene® Abbvie, Ludwigshafen, Germany). After one week of recovery, body mass, fur index, and body temperature were monitored weekly until 15±2 weeks of SP exposure. Body temperature was recorded in 3 mins intervals. Torpor was defined as reduction of body temperature below 32°C for at least 30 min. The animals were sacrificed by CO_2_ inhalation during the light-phase, either during hypothermia (HT) conditions of a torpor bout or during normothermia (NT) on a day without torpor (for details see S1 Fig and [3]).

### Food restriction under long photoperiod (LP-FR)

Twenty female Djungarian hamsters (age of 14 ± 2 months) were used in this experiment, as illustrated in Fig 1. To assess baseline body weight and daily food intake, the hamsters were monitored over two weeks and the week after, half of the hamsters also received an intraperitonealtransmitter implant (TA11TA-F10). The hamsters were fed *ad libitum* for an additional week after surgery. Afterwards, the animals were divided into two weight-matched groups: the first group comprised ten hamsters that were fed *ad libitum* and served as control group (LP-AL), and the remaining ten implanted hamsters were subjected to a long-term food restriction (LP-FR) (Fig 1). In the first week of food restriction, the 10 LP-FR hamsters were restricted by 70% of their individual daily *ad libitum* food intake. For additional four weeks, food restriction was reduced to 40% of the hamsters’ baseline daily food intake. Supplemental feeding (+10%) was provided when an animal had lost more than 25% of its baseline body weight. During the following five weeks, body weight and daily food intake of all 20 hamsters was regularly measured in the LP-AL group. In the LP-FR group, body temperature was recorded in three-minute-intervals to detect potential fasting-induced torpor bouts, characterized by a body temperature below 32°C for at least 30 minutes. At the end of week five, all hamsters were killed by CO_2_ inhalation for tissue sampling during the light phase and thus the hamsters’ resting phase. No hamster expressed torpor during sacrifice.

### Tissue sampling and storage

Blood samples were obtained by puncture of the right cardiac ventricle and stored at 4°C in EDTA-coated microvettes (Sarstedt, Nümbrecht, Germany). Samples were centrifuged for 10 minutes at 4°C and 2000 rpm, plasma and pellet were stored separately at -80°C. Additionally, brain, liver, heart, and small intestine were dissected, snap frozen over dry ice, and stored at -80°C until further use in this and other studies (see Data availability).

### Measurements of plasma and liver iron content

The plasma iron concentration was measured with the Thermo Scientific iron kit (ref: 981236) using 10 μl of plasma and a serial dilution of an iron atomic absorption standard solution (Sigma, Germany). The non-heme iron content of the liver was measured by a colorimetric assay as previously described [22] and the results are expressed in micrograms of iron per gramm of dry tissue weight.

### Measurements of liver trace metals

Determination of trace metals in liver tissue was achieved by total reflection X-ray fluorescence spectrometry (TXRF) of acidic homogenates. First, liver tissue was thawed, weighed and then 1 mL of subboiled nitric acid (HNO3, 63% AnalR Normapur, VWR International GmbH, Darmstadt, Germany) was added. The samples were vigorously shaken at 2,500 rpm for 60 s to partially digest the biological matrix. Subsequently, the resulting suspensions were treated in an ultrasonic bath at 40°C for 15 min, followed by another mixing step for 60 s at 2,500 rpm. Finally, 10 μL titanium standard solution (100 mg/L in 2% HNO3) were added as an internal standard for calibration of TXRF measurement, and the mixture was vortexed for another 60 s at 2,500 rpm. An aliquot of 10 μL sample suspension was applied onto a TXRF sample carrier and measurement was performed using high-efficiency module S2 Picofox (Bruker Nano GmbH, Berlin, Germany) equipped with Mo X-ray tube. For each sample, three replicate sample carrier were prepred and measured (n = 3). All details of biological sample preparation, TXRF measurement, and instrumentation were previously described [23].

### RNA isolation, reverse‐transcription, and real‐time PCR

The gene expression levels of Hepcidin (*Hamp*) and Transferrin Receptor 1 (*Tfr1*) were measured by real-time PCR. Total RNA was isolated from the liver tissue using TRIzol® (Invitrogen) according to the manufacturer’s instructions. RNA quality and quantity were controlled using the Nanodrop 2000 system (Thermo Scientific, Waltham, MA, USA). Reverse transcription was performed using 2 μg of total RNA following the manufacturer’s instructions. qPCR was carried out in 10 μL of reaction volume using SYBR Green I Dye (Invitrogen, Carlsbad, CA, USA) on ABI ViiA‐7 system (Applied Biosystems, Foster City, CA, USA). All data were normalized to *18S* ribosomal housekeeping gene. The following primer sequences were used: *18S* (Forward: 5’-GCTCCTCTCCTACTTGGATAACTGTG-3’ and reverse: 5’-CGGGTTGGTTTTGATCTGATAAATGCA-3’), *Hamp* (Forward: 5’-TGTTTCTGGATCCCTGCTCC-3’ and reverse: 5’-TTGGACCGACACACAGACAG-3’), *Tfr1* (Forward: 5’-ACATGGCAGGCACACAATTA-3’ and reverse: 5’-TGCCCTCTGGAGACTTCCTAA-3’).

### Data analysis and statistics

Data were analysed using GraphPad Prism software (GraphPad Software, La Jolla, CA, USA). Results are given as mean ± SD. Individual values of iron and trace element levels were normalised to final body weight (g). Torpor incidence was calculated for each hamster and expressed as the relative number of torpor bouts per observation interval. Data were tested for normal distribution using the Shapiro-Wilk test. Comparisons between two groups were tested for significance using the t-test for normally distributed data and the Mann-Witney test for non-normally distributed data. Correlations were determined using the Spearman rank test for non-normally distributed data or Pearson for normally distributed data. Statistically significant differences are indicated by *p <* .*05* (*), *p <* .*01* (**), and *p <* .*005* (***).

## Results

In this study, we investigated the effects of seasonal acclimation and food restriction on the metabolism of iron and other trace elements under laboratory conditions. The results are presented in two contexts. First, the long- and short-photoperiod groups of hamsters (LP-AL and SP-AL, respectively) were used to analyse potential effects of photoperiod and consequent long-term metabolic acclimation (such as body weight change and spontaneous daily torpor expression) on hamsters’ liver iron levels. In the second part we have addressed the effect of food restriction and fasting-induced torpor expression on the metabolism of iron and other trace elements.

### Effect of photoperiodic changes on iron levels in hamsters

**Fig 2:**
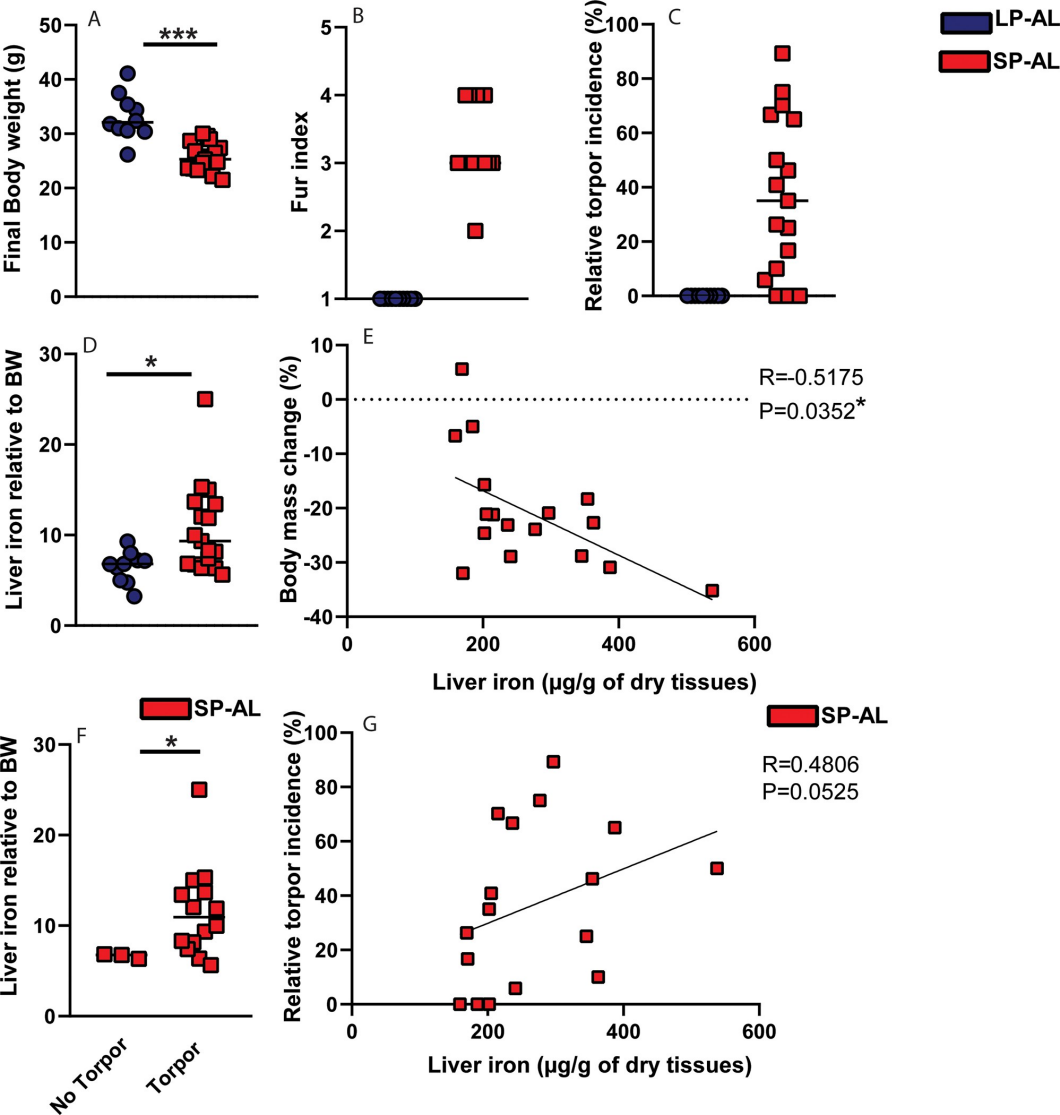
Effect of photoperiodic changes on torpor incidence and hepatic iron levels in hamsters. Djungarian hamsters were exposed to a long photoperiod *ad libitum* (LP-AL; 16h light and 8h dark; *n* = 10) or short photoperiod *ad libitum* (SP-AL; 8h light and 16h dark; *n* = 18). At the end of the experiments, hamsters were sacrificed and (A) Body weight (g), (B) Fur index and (C) torpor incidence (%) were measured. (D-E) Non-heme liver iron content, expressed in μg iron per gram dried liver tissue, was normalized to final body weight (BW) in both groups and correlated to body mass loss (%) in SP-AL group. (F-G) Non-heme iron content in the liver, expressed as μg iron per gram dried liver tissue, was normalized to final body weight (BW) in SP-AL groups with or without torpor expression (G) and correlated to the relative torpor incidence. All data are shown as mean ±SD. Statistically significant differences are indicated by p < .05 (*), p < .005 (***).

The seasonal acclimation process can be successfully mimicked in laboratory conditions [3,24] where hamsters are housed to long-photoperiod (LP) of 16h light and 8h dark during summer time, and short-photoperiod (SP) of 8h light and 16h dark during winter time. By using this approach (Fig 1), we observed that hamsters in the short-photoperiod (SP-AL) group experienced a significant body weight loss by approximately 30% (Fig 2A), and an increase in fur index up to 4 points (Fig 2B), demonstrating that hamsters have acclimated to short-photoperiod exposure. Regarding spontaneous daily torpor, 14 out of 17 hamsters in the SP-AL group expressed torpor with relative incidence between 6–89% and the remaining three hamsters did not express torpor (Fig 2C). No torpor expression was present in LP-AL group (Fig 2C). Within the SP-AL group, nine hamsters were sacrificed during a torpor bout when their final body temperature was below 32°C (referred to as ‘Hypothermic’, S1A and S1B Fig); 5 hamsters, expressing history of torpor bouts before sacrifice, and 3 non-torpid animals, were sacrificed during their resting phase on a day without torpor expression with a final body temperature above 32°C (referred to as ‘Normothermic’, S1A and S1B Fig).

In regard to iron status, we measured a significant increase of non-heme iron in the livers of hamsters undergoing short photoperiod acclimation and this effect was enhanced when liver iron content was normalized to body weight (1.7-fold; *p* < 0.011) (Fig 2D and 2E). Importantly, changes in liver iron content correlated positively with torpor incidence (Fig 2F and 2G), implying that acclimation process and the history of the torpor expression associate with iron sequestration in the liver. There was no difference in liver iron levels between hamsters that were sacrificed during a torpor bout (‘Hypothermic’) and hamsters that expressed torpor but were sacrificed during their resting phase (‘Normothermic’) (S1C Fig). We conclude that only those hamsters that acclimated and expressed torpor showed increased iron deposition in the liver, the finding that was not observed in non-torpid animals (Fig 2F and 2G).

### Effects of food restriction and torpor incidence on systemic iron metabolism

We next questioned whether food restriction, hence forced body weight reduction, affects iron metabolism to similar extent as voluntary seasonal body weight reduction. To this end, we used a previously established regime where hamsters were subjected to 5 weeks of moderate food restriction (Fig 1) [11].

**Fig 3:**
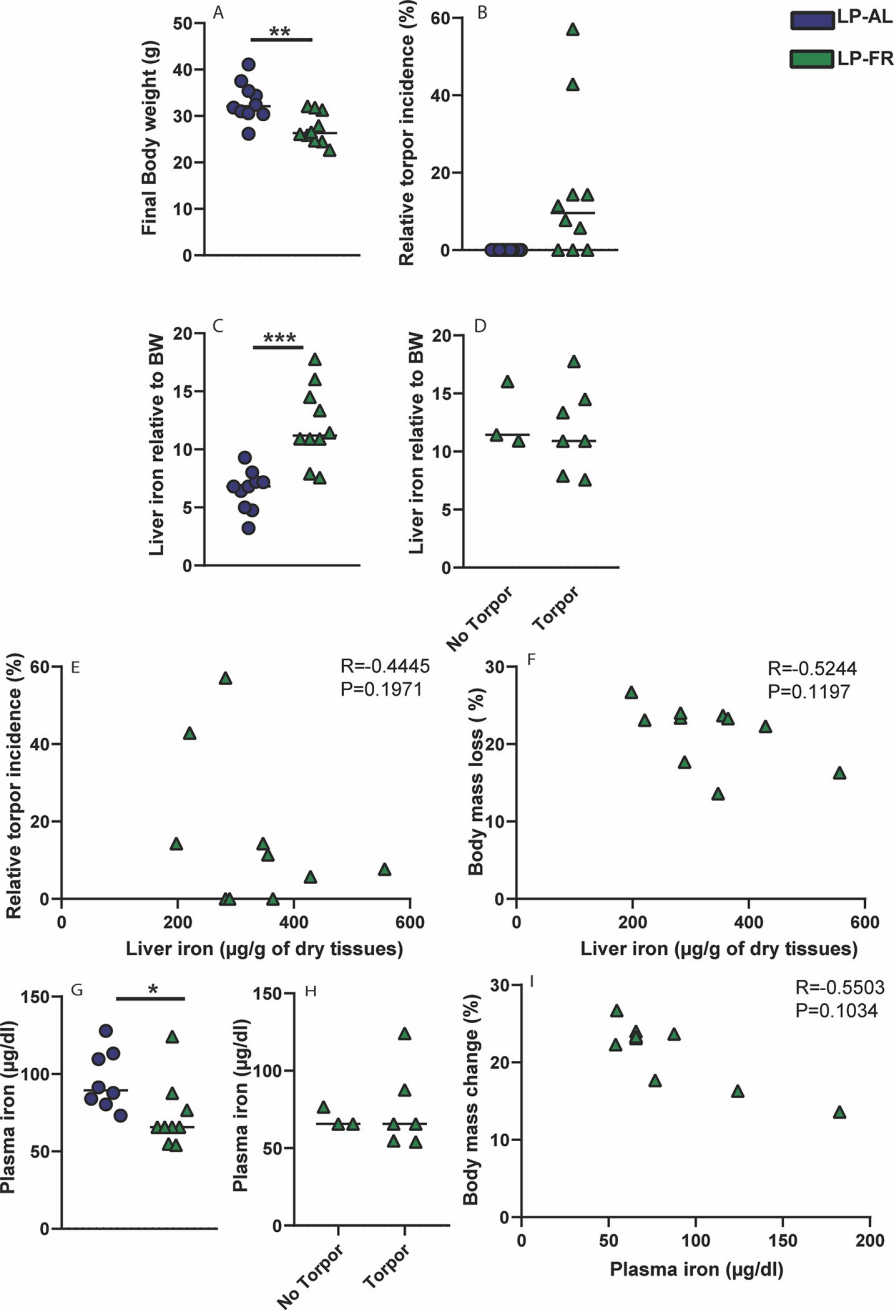
Effect of food restriction on torpor incidence and systemic iron levels. (A) Djungarian hamsters were exposed to a long photoperiod (LP; 16h light and 8h dark) and subjected to either food restriction for 5 weeks (LP-FR, *n* = 10) or *Ad libitim* (LP-AL) (*n* = 10). At the end of the experiments, hamsters were sacrificed and (A) final body weight (g) and (B) The relative torpor incidence (%) were measured in both groups. (C-F) Non-heme iron content in the liver, expressed as μg iron per gram dried liver tissue, was normalized to final body weight (BW) and correlated to the expression of torpor and the percentage of body mass loss. (G-I) Plasma iron level (μg/dl) in LP-AL and LP-FR groups depending on the torpor expression and its correlation to body mass loss (%) in LP-FR group. All data are shown as mean ±SD. Statistically significant differences are indicated by p < .05 (*), p < .01 (**), p < .005 (***).

We first show that food restriction (LP-FR) resulted in approximately 21% body weight reduction when compared to the *ad libitum* group (LP-AL) (Fig 3A). In addition to weight loss, torpor incidence was recorded and ranged from 0 to 57% in LP-FR group, whereby 3 out of 10 hamsters did not express torpor (Fig 3B).

Non-heme iron content was increased in the livers of hamsters in LP-FR group by 1.54-fold when compared to the LP-AL group and the effect was more pronounced when the liver iron content was normalized to the body weight resulting in approximately 2-times higher iron levels in LP-FR than in LP-AL group (Fig 3C). Importantly, increased hepatic iron levels were present in both, the no torpor and torpor LP-FR subgroup, thus independent of torpor incidence (Fig 3D and 3E) and independent of the body mass loss (Fig 3F). By contrast, iron levels in the blood were significantly decreased in LP-FR group when compared to the *ad libitum* fed group (LP-AL) (Fig 3G); however, there was no correlation with torpor use (Fig 3H and 3I). We conclude that food restriction leads to hypoferremia and iron sequestration in the liver and that this occurs independently of the torpor incidence.

**Fig 4:**
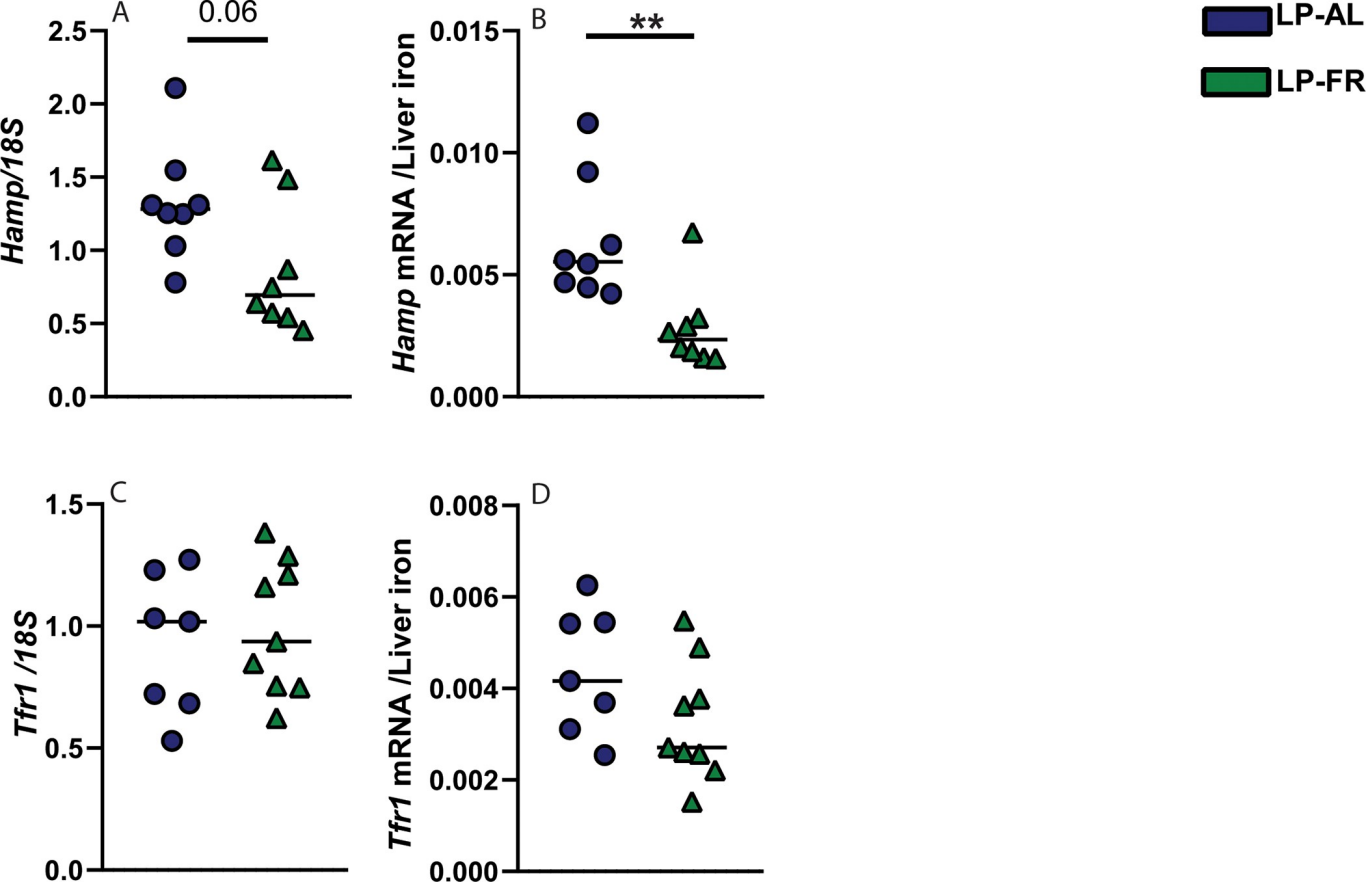
Effect of food restriction and torpor incidence on iron regulatory genes. (A) Hepatic mRNA expression of (A, B) hepcidin (*Hamp*) and of (C, D) transferrin receptor 1 (*Tfr1*) were quantified by real-time PCR and normalized to 18S expression and to non-heme liver iron content. All data are shown as mean ±SD. Statistically significant differences are indicated by p < .05 (*), *p <* .*01* (**).

To clarify the possible mechanism by which fasting and torpor regulate the expression of iron regulatory genes, we studied the effect of fasting on the mRNA expression of hepatic hepcidin (*Hamp*) and transferrin receptor 1 (*Tfr1)*. Hepcidin mRNA levels were marginally statistically different between LP-AL and LP-FR groups (p = 0.06), and when normalized to liver iron content, hepcidin levels were 2.26-fold lower in LP-FR; *p* = 0.004 (Fig 4A and 4B). In contrast to hepcidin, the expression of *Tfr1* did not change, indicating that iron import is likely not affected under conditions of long-term food restriction (Fig 4C and 4D).

### Effect of food restriction and torpor incidence on the distribution of trace elements in the liver

**Fig 5:**
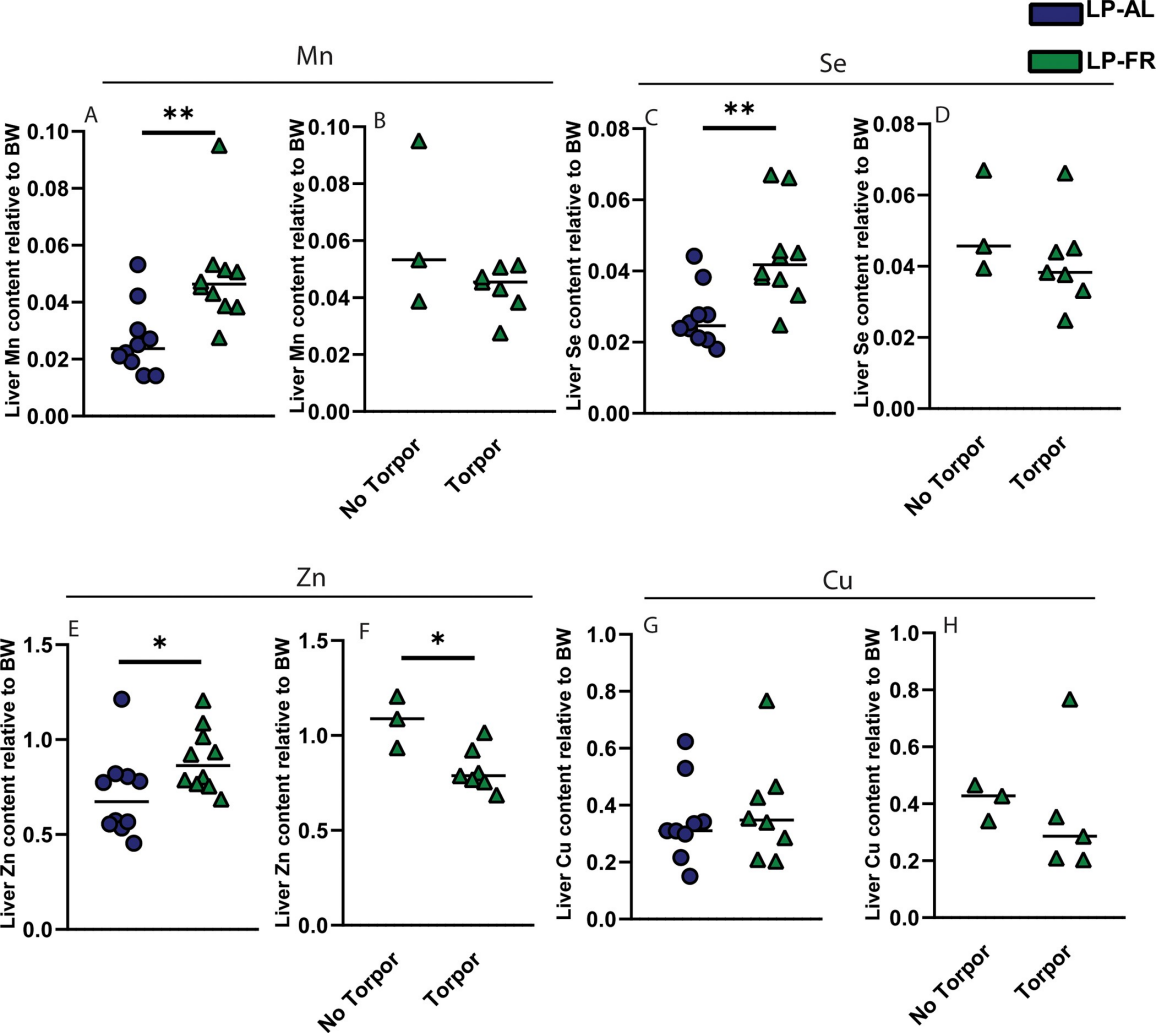
Effect of food restriction on distribution of trace elements in the liver. (A) The levels of (A,B) Manganese (Mn), (C,D) Selenium (Se), (E,F) Zinc (Zn), and (G,H) Copper (Cu) (ng/ gram liver tissues) were measured by TXRF and normalized to body weight (BW) of hamsters in LP-AL and LP-FR group, and depending on the torpor expression in the latter. All data are shown as mean ±SD. Statistically significant differences are indicated by *p <* .*05* (*), *p <* .*01* (**).

Given the seasonal acclimation and food restriction regime resulted in iron sequestration in the liver, we next investigated the levels of other essential trace elements using the total reflection X-ray fluorescence method (TXRF) [23]. We show that liver manganese (Mn) content was significantly increased in LP-FR group (Fig 5A and 5B). Similar to Mn content, selenium content (Se) was increased in the LP-FR group compared to LP-AL (Fig 5C and 5D) and a significant difference was also observed for the levels of zinc (Zn) between the LP-AL and LP-FR groups (Fig 5E and 5F), whereas no differences were found between the groups with respect to copper (Cu) levels (Fig 5G and 5H). Based on these data, we conclude that hamsters tend to sequester Mn, Se and Zn, in addition to iron, in the liver during the food restriction period; however this increase does not correlate with body mass change or torpor expression (Table 1).

**Table 1:** Summary of potential correlations between relative body mass change (%) as well as relative torpor incidence (%) and the levels of trace elements in the liver (absolute values) of the food-restricted (LP-FR) hamster group.

LP-FR	Liver manganese content (ng/mg)	Liver selenium content (ng/mg)	Liver zinc content (ng/mg)	Liver copper content (ng/mg)
**Body mass change (%)**	R = -0.1724 p = 0.6339	R = -0.1651 p = 0.6486	R = -0.5465 p = 0.1021	R = -0.3810 p = 0.3599
**Torpor incidence (%)**	R = -0.5293 p = 0.1195	R = -0.2893 p = 0.4131	R = -0.5847 p = 0.0810	R = -0.4880 p = 0.2271

## Discussion

Hamsters, express torpor when facing unfavorable environmental conditions such as seasonal change and/or food restriction. To acclimate during time of short photoperiod (SP), hamsters undergo several metabolic adaptation and express torpor to highly individual levels. Our study demonstrates that these changes are associated with iron deposition in the liver. Importantly, only hamsters that acclimated and expressed torpor during the acclimation process showed increased hepatic iron levels implicating that iron sequestration in the liver positively associates with the incidence of torpor expression.

Moreover, hamsters also adapted their metabolic profile once facing high nutrient and energy deficits, and this adaptation is highly similar to short-photoperiod acclimation. Namely, by comparing a forced torpor state, that occurred during food restriction (LP-FR), with spontaneous torpor during short photoperiodic acclimation under our laboratory conditions (SP-AL), we see similar features between the two types of torpor; this includes 1) an overall decrease in body mass in the LP-FR group and in SP-AL (Supp. 2A), 2) similar torpor occurence (Supp. 2B,C), and 3) similar increase in hepatic iron levels (Supp. 2D). Importantly, food deprivation in hamsters led to the development of hypoferremia and similar findings were recently observed in mice and rats on mild to moderate nutrient deficit suggesting that food restriction contributed to lowering circulating iron levels and promoting iron sequestration in tissues such as the liver [25]. This effect associated with lower hepcidin expression. We speculate that low hepcidin may increase iron export from ferroportin-expressing cells and subsequent redistribution of iron towards the liver (hepatocytes) where iron is taken up by transporters other than Tfr1.

Taken together, our data clearly indicate that the consequence of overall metabolic adaptation to short photoperiod and food restriction include torpor expression and a disturbance in liver iron metabolism leading us to propose that changes in systemic iron levels may be a component of the adaptation strategy. Restricting iron availability might have evolved as a long-term compensatory mechanism which allowed hamsters to cope with drastic environmental challenges and physiological demands, and to protect animals against pathogens that require iron for their growth. Interestingly, long-term hibernation has been reported to disrupt iron metabolism in other animals such as brown bears, ground squirrels, and Svalbard reindeer [26–28]. Although torpor expression contrasts hibernation, which is generally characterized by long torpor bouts lasting several days to weeks usually interrupted by brief normothermic interbout arousals, the underlying molecular mechanisms of iron sequestration in the liver during short photoperiod acclimation, food deprivation, and hibernation, are likely to share some common molecular signatures, and warrant further investigation.

In addition to iron, we show that other trace elements such as manganese, selenium and zinc were increased in the livers of hamsters during food deprivation. Most of these essential trace elements have immunomodulatory and antimicrobial effects and generally serve as enzyme cofactors, antioxidants, and/or anti-inflammatory agents and play an important role in oxidative stress and redox potentials [29,30]. Higher levels of trace elements in the liver, and possibly in other tissues, may be essential during food deprivation and torpor state for enhancing antioxidant defence capacity to scavenge excess ROS and maintain cell redox homeostasis in hibernating mammals. Indeed, hibernators such as the Daurian ground squirrel (*Spermophilus dauricus*) rely on the upregulation of several antioxidant enzymes to avoid oxidative stress during hibernation [31].

Collectively, our study illustrates that liver iron levels are related to torpor expression during the course of the acclimation of hamsters to challanging environmental conditions. Further investigations are required to address the systemic, cellular and molecular changes in the metabolism of iron and other trace elements in hibernating and torpid animals and their role in various adaptation processes/mechanisms.

## Supporting information

S1 Fig Effect of short photoperiod on torpor incidence and hepatic iron levels in normothermic and hypothermic hamsters. (A) Terminal body temperature (°C) and (B) the relative torpor incidence (%) of SP-AL hamsters based on the normothermic and hypothermic status. (C) Non-heme liver iron content (μg iron per gram dried liver tissue) was normalized to final body weight (g) in SP-AL hamsters based on the normothermic and hypothermic status. All data are shown as mean ±SD.
(TIF)

S2 Fig Comparison between spontaneous and forced torpor state. One group of Djungarian hamsters was exposed to a long photoperiod under moderate long-term food-restriction (LP-FR; 16h light and 8h dark; *n* = 10), while the other group was exposed to a short photoperiod under *ad libitum* (SP-AL; 8h light and 16h dark; *n* = 8). Comparison between (A) the final body weight, (B) torpor incidence (%), (C) terminal body temperature (°C), and (D) the non-heme liver iron content (μg iron per gram dried liver tissue) normalized to final body weight (g). All data are shown as mean ±SD.
(TIF)
