# Supplementary figures and images for: Seasonal and fasting induced changes in iron metabolism in Djungarian hamsters
Source: PLoS One. 2023 Nov 6;18(11):e0293971. doi: 10.1371/journal.pone.0293971 (PMC10627461; doi:10.1371/journal.pone.0293971)

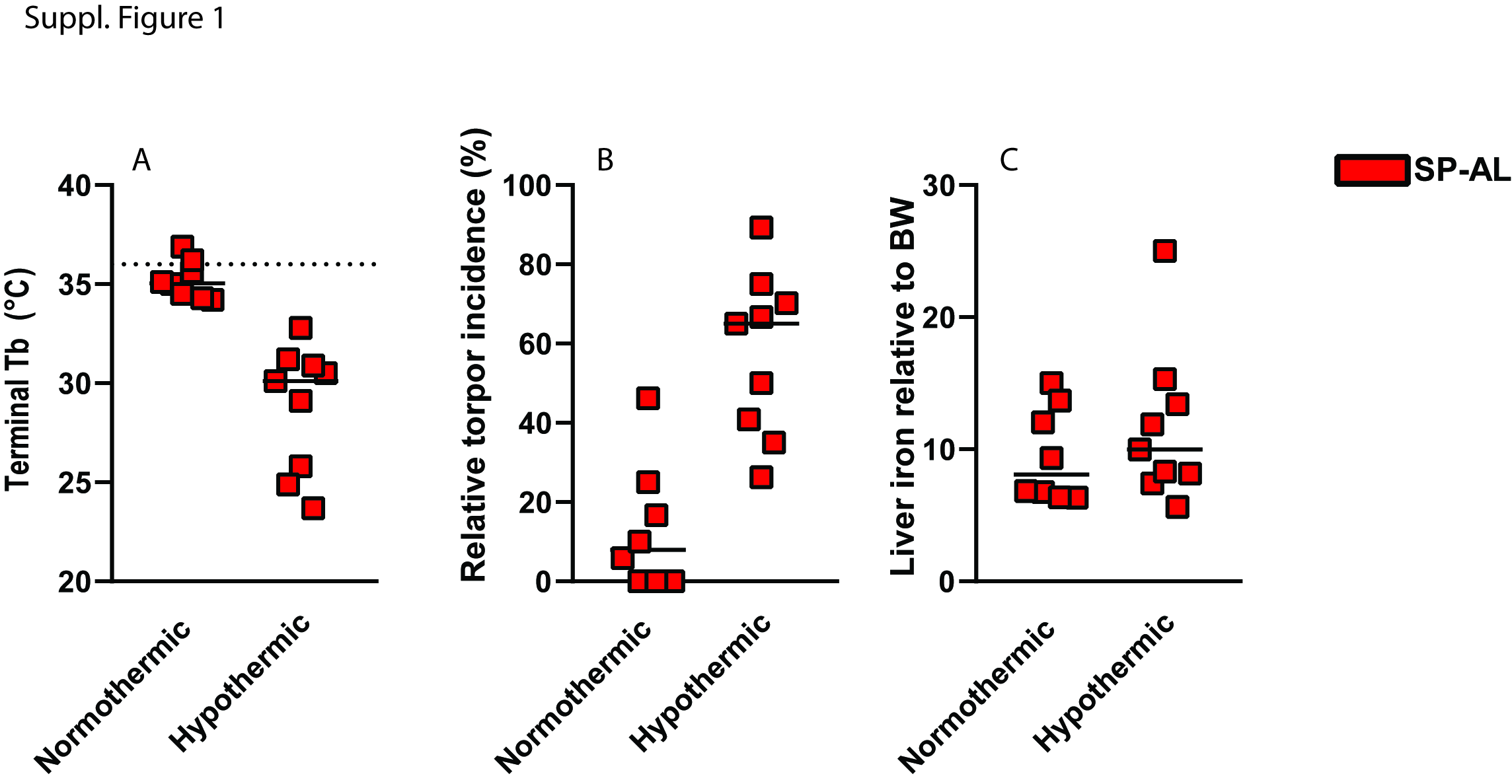

Supplement: S1 Fig — (A) Terminal body temperature (°C) and (B) the relative torpor incidence (%) of SP-AL hamsters based on the normothermic and hypothermic status. (C) Non-heme liver iron content (μg iron per gram dried liver tissue) was normalized to final body weight (g) in SP-AL hamsters based on the normothermic and hypothermic status. All data are shown as mean ±SD. (TIF) [file pone.0293971.s001.tif]

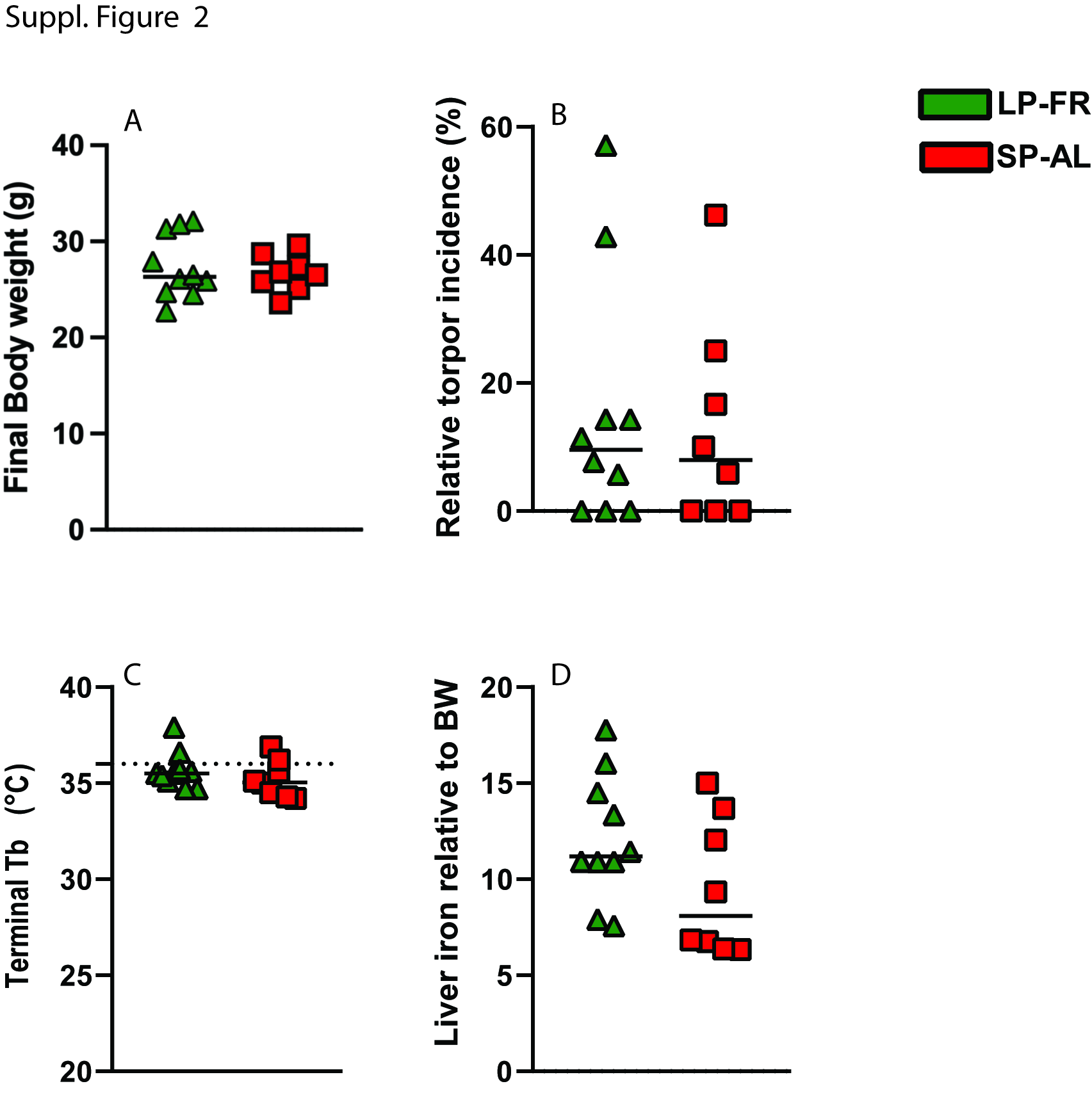

Supplement: S2 Fig — One group of Djungarian hamsters was exposed to a long photoperiod under moderate long-term food-restriction (LP-FR; 16h light and 8h dark; n = 10), while the other group was exposed to a short photoperiod under ad libitum (SP-AL; 8h light and 16h dark; n = 8). Comparison between (A) the final body weight, (B) torpor incidence (%), (C) terminal body temperature (°C), and (D) the non-heme liver iron content (μg iron per gram dried liver tissue) normalized to final body weight (g). All data are shown as mean ±SD. (TIF) [file pone.0293971.s002.tif]
